# Supplementary material for: In-planta Gene Targeting in Barley Using Cas9 With and Without Geminiviral Replicons
Source: Front Genome Ed. 2021 Jun 15;3:663380. doi: 10.3389/fgeed.2021.663380 (PMC8525372; doi:10.3389/fgeed.2021.663380)
Supplement: Supplementary Figure 1 — Gel showing sensitivity obtained in F1/R1 PCR screen setup. Construct D was transformed into barley and DNA extracted from a regenerated plant (transgene copy number 1) and quantified by Qubit fluorescence. Serial dilutions were made of this DNA for subsequent PCR. The copy number of transgene D are shown for each lane. The limit of detection is around 40 copies of the target. [file Presentation_1.PPTX]

## Slide 1
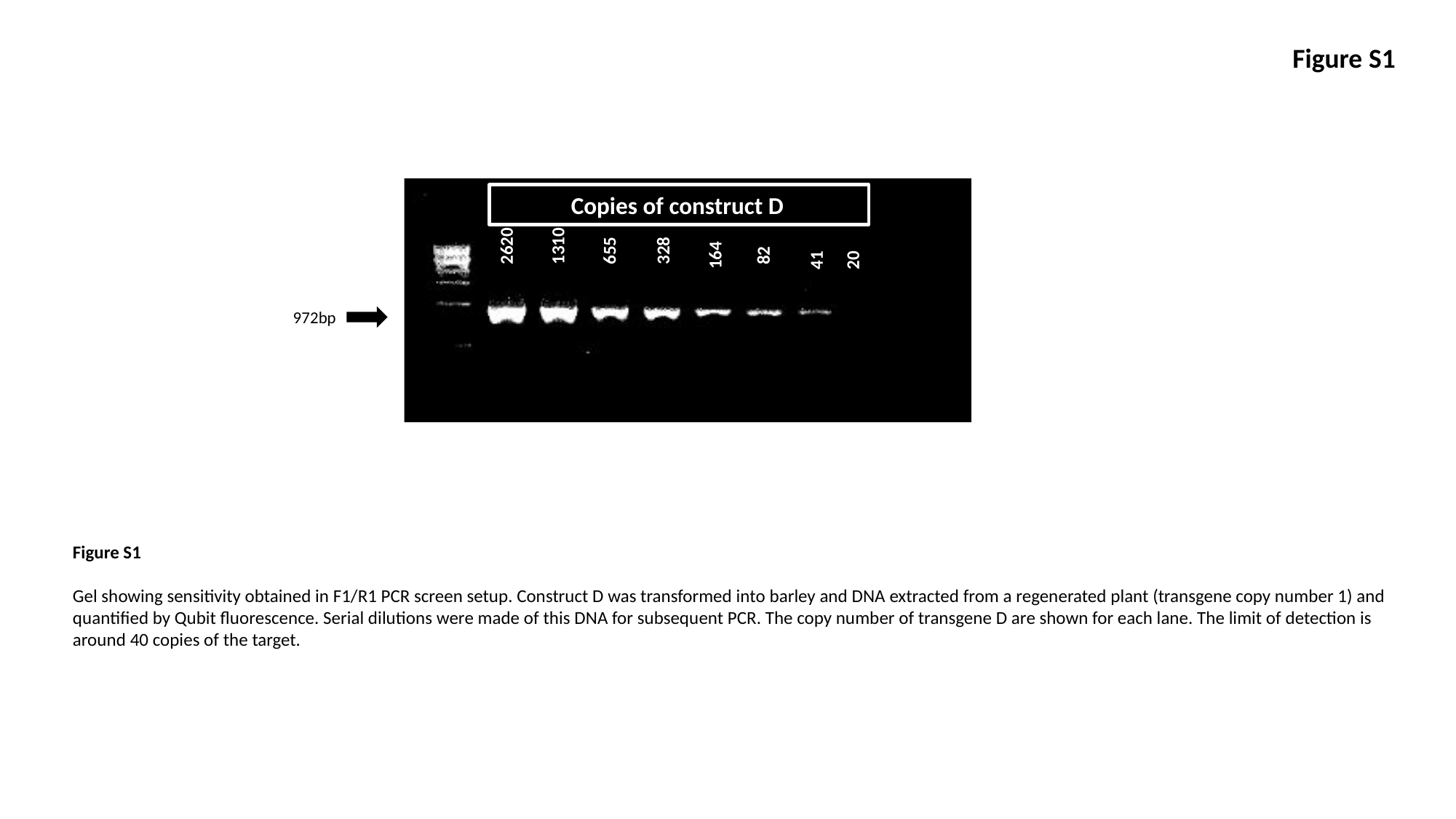

Figure S1
 Copies of construct D
2620
1310
655
328
164
82
41
20
972bp
Figure S1
Gel showing sensitivity obtained in F1/R1 PCR screen setup. Construct D was transformed into barley and DNA extracted from a regenerated plant (transgene copy number 1) and quantified by Qubit fluorescence. Serial dilutions were made of this DNA for subsequent PCR. The copy number of transgene D are shown for each lane. The limit of detection is around 40 copies of the target.
