# Supplementary material for: In-planta Gene Targeting in Barley Using Cas9 With and Without Geminiviral Replicons
Source: Front Genome Ed. 2021 Jun 15;3:663380. doi: 10.3389/fgeed.2021.663380 (PMC8525372; doi:10.3389/fgeed.2021.663380)
Supplement: Supplementary Figure 2 — Copies of repair template plasmid per target site. Thirty ng of wild type barley DNA was mixed with serial dilutions of plasmid DNA containing the repair template. The lane numbers represent the copies of repair template: target site ratio. Positive control was a construct D line. The 1047 bp band in lane 736641 was excised, purified and sequenced. [file Presentation_2.PPTX]

## Slide 1
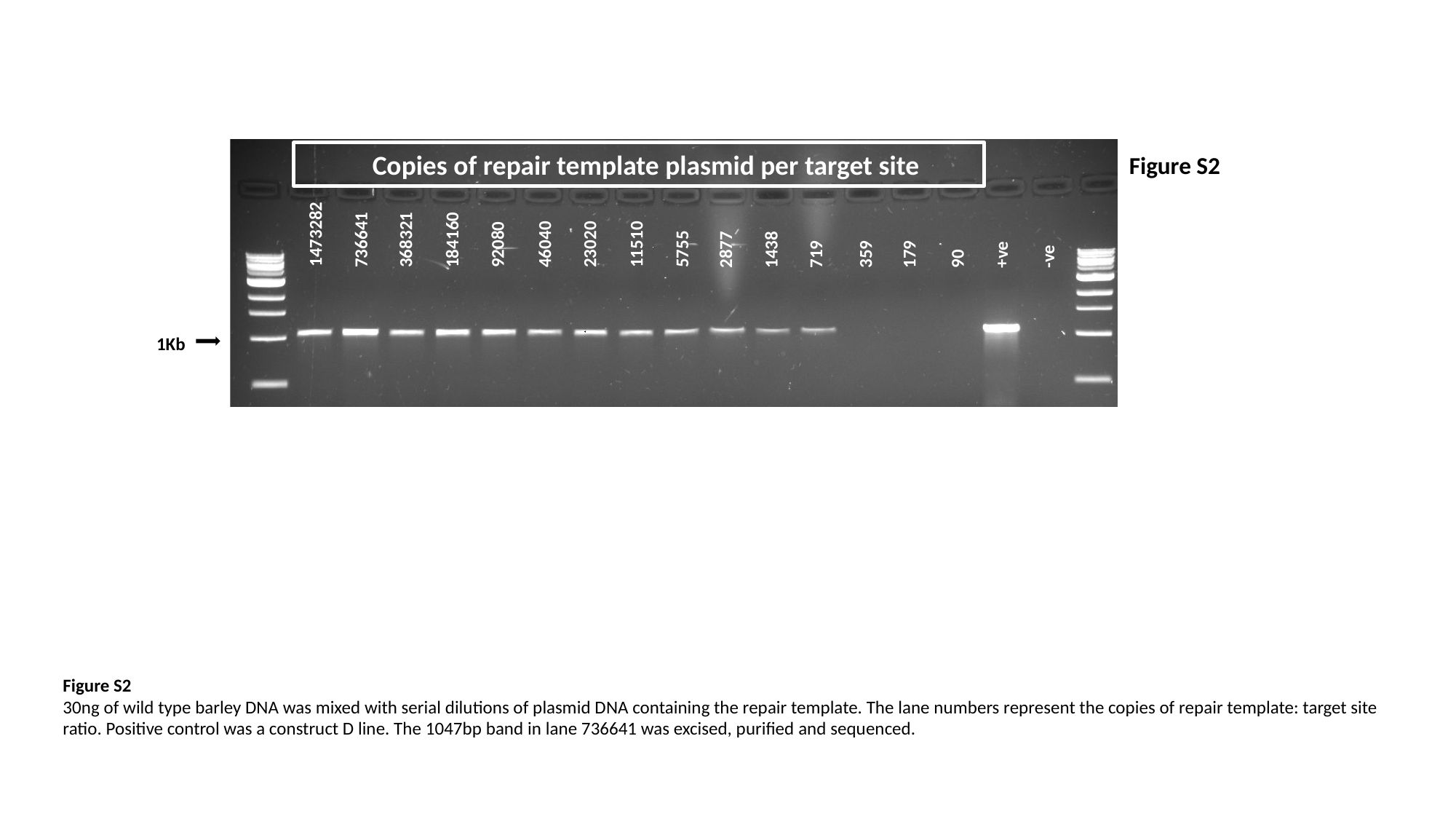

Copies of repair template plasmid per target site
1473282
736641
368321
184160
92080
46040
23020
11510
5755
2877
1438
719
359
179
90
+ve
-ve
1Kb
Figure S2
Figure S2
30ng of wild type barley DNA was mixed with serial dilutions of plasmid DNA containing the repair template. The lane numbers represent the copies of repair template: target site ratio. Positive control was a construct D line. The 1047bp band in lane 736641 was excised, purified and sequenced.
