# Supplementary material for: In-planta Gene Targeting in Barley Using Cas9 With and Without Geminiviral Replicons
Source: Front Genome Ed. 2021 Jun 15;3:663380. doi: 10.3389/fgeed.2021.663380 (PMC8525372; doi:10.3389/fgeed.2021.663380)
Supplement: Supplementary Table 2 — Sequences of GT events for lines 2158-9-1, 2158-14-1, 1826-5-2, and 1826-8-1 showing F1/R1 (T0), F2/R2 (T0), and F1/R3 (T1) products. [file Table_2.DOCX]

**Supplementary Table 2**

Sequences of GT events for lines 2158-9-1, 2158-14-1, 1826-5-2, 1826-8-1

F1/R1 T0 products

agaggttagcctttgtagatggtattgggcttatattgggttccatgggcggagtttgtttttcgaacaccagatttataggcatggtaaatcaaatgtttttatggcacatatgtgttcgctgaggatggcaagtttagttaacaagcatgtcaaatttgactcaatttattttttgccaagaaattgtcgtgcttgcaaacttaatttgccaccctgacgccaatataaattgccatacaaaatgtttgatttgccatgcctaattttttagcatttttgtttcttttatcattagccattatcttcttctttttaaaatcttatagtatgcaaatctaataagattcctggcgagttattaagaaaaccagattctcattattttttcctttgcaaaaaagagaagattctctctctctcttacaacgattctcattctccggctcaaaaaaaagtttgtttctcattctcttcctgcttaatgcaatcggtattttttttttgaggggaacttaatgcaatcagtagagtgcttgcctcgttgctggaaaaagaattgatgatccatgtgatttaacgagaaaaacaaagtccgcccatggtgcccaatattttaggcccagttgggatggtagaacctgctgctggagccagctccagttttgttcgccgaatgccgagtcccggcgcccagggatggctataaataagcgagctcccgtgtccttgtgtacttgtaaaatctgtgctccctgcccaccgctctcccctcggttcccacgcgccaaaacattccaacgtggagacaagaagcagcatagcgtgacaacgagggagggagccatggacgtgaccatggaggacgtgatggtgagcaagggcgaggaggataacatggccatc

F2/R2 T0 products

gaccacctacaaggccaagaagcccgtgcagctgcccggcgcctacaacgtcaacatcaagttggacatcacctcccacaacgaggactacaccatcgtggaacagtacgaacgcgccgagggccgccactccaccggcggcatggacgagctgtacaagctccacggcctgatcgagtccatgctctgcgatgacactctcatcggcacgcccgagcccgacgagcacccagacccagccatgttcacggacggcccctgctactccaacggctccgacccgagcagcaccaccacgacgaacccgggcacgcccgtgcagcacgacgacgacctgccgcaggactgcaatcccgagaagggactccggctgcttcacctgctcatggccgccgccgaggcgctctccggcccgcacaagagccgggagctggcacgggtgatattggttcggctcaaggagatggtctccagcaccagcggcaacgctgccgcgtccaacatggagcgcctcgccgcccacttcaccgacgcgctccaggggctcctcgatgggtcccactccgtcgctgggaccagcaggcaggccgcatcccaccaccacagcaccggcgacgtgttgacggcattccagatgctccaggacatgtcgccctacatgaagttcggccacttcaccgcgaaccaggcgatcctggaggcggtggcgggcgaccggcgcgtccacatcgtggactacgacctcgccgagggcatccagtgggcgtccctgatgcaggctatgacatcacgacccgatggcgtgtcgcctccgcacctgcgtatcaccgccatcacgcggagtggcgggggcggcgcgcgggcagtccaggaggccggacggcgcctcgcggccttcgcggggtccatcgggcagcccttctcgttcggacattgccgtctggactcggacgagaggttccggccggcgaccgtcaggatggtcaagggggagacgctcgtggcca

F1/R3 T1 products

agaggttagcctttgtagatggtattgggcttatattgggttccatgggcggagtttgtttttcgaacaccagatttataggcatggtaaatcaaatgtttttatggcacatatgtgttcgctgaggatggcaagtttagttaacaagcatgtcaaatttgactcaatttattttttgccaagaaattgtcgtgcttgcaaacttaatttgccaccctgacgccaatataaattgccatacaaaatgtttgatttgccatgcctaattttttagcatttttgtttcttttatcattagccattatcttcttctttttaaaatcttatagtatgcaaatctaataagattcctggcgagttattaagaaaaccagattctcattattttttcctttgcaaaaaagagaagattctctctctctcttacaacgattctcattctccggctcaaaaaaaagtttgtttctcattctcttcctgcttaatgcaatcggtattttttttttgaggggaacttaatgcaatcagtagagtgcttgcctcgttgctggaaaaagaattgatgatccatgtgatttaacgagaaaaacaaagtccgcccatggtgcccaatattttaggcccagttgggatggtagaacctgctgctggagccagctccagttttgttcgccgaatgccgagtcccggcgcccagggatggctataaataagcgagctcccgtgtccttgtgtacttgtaaaatctgtgctccctgcccaccgctctcccctcggttcccacgcgccaaaacattccaacgtggagacaagaagcagcatagcgtgacaacgagggagggagccatggacgtgaccatggaggacgtgatggtgagcaagggcgaggaggataacatggccatcatcaaggagttcatgcgcttcaaggtgcacatggagggctccgtgaacggccacgagttcgagatcgagggcgagggcgagggccgcccctacgagggcacccagaccgccaagctgaaggtgaccaagggtggccccctgcccttcgcctgggacatcctgtcccctcagttcatgtacggctccaaggcctacgtgaagcaccccgccgacatccccgactacttgaagctgtccttccccgagggcttcaagtgggagcgcgtgatgaacttcgaggacggcggcgtggtgaccgtgacccaggactcctccctgcaggacggcgagttcatctacaaggtgaagctgcgcggcaccaacttcccctccgacggcccagtaatgcagaagaaaaccatgggctgggaggcctcctccgagcggatgtaccccgaggacggcgccctgaagggcgagatcaagcagaggctgaagctgaaggacggcggccactacgacgctgaggtcaagaccacctacaaggccaagaagcccgtgcagctgcccggcgcctacaacgtcaacatcaagttggacatcacctcccacaacgaggactacaccatcgtggaacagtacgaacgcgccgagggccgccactccaccggcggcatggacgagctgtacaagctccacggcctgatcgagtccatgctctgcgatgacactctcatcggcacgcccgagcccgacgagcacccagacccagccatgttcacggacggcccctgctactccaacggctccgacccgagcagcaccaccacgacgaacccgggcacgcccgtgcagcacgacgacgacctgccgcaggactgcaatcccgagaagggactccggctgcttcacctgctcatggccgccgccgaggcgctctccggcccgcacaagagccgggagctggcacgggtgatattggttcggctcaaggagatggtctccagcaccagcggcaacgctgccgcgtccaacatggagcgcctcgccgcccacttcaccgacgcgctccaggggctcctcgatgggtcccactccgtcgctgggaccagcaggcaggccgcatcccaccaccacagcaccggcgacgtgttgacggcattccagatgctccaggacatgtcgccctacatgaagttcggccacttcaccgcgaaccaggcgatcctggaggcggtggcgggcgaccggcgcgtccacatcgtggactacgacctcgccgagggcatccagtgggcgtccctg
