# Supplementary material for: In-planta Gene Targeting in Barley Using Cas9 With and Without Geminiviral Replicons
Source: Front Genome Ed. 2021 Jun 15;3:663380. doi: 10.3389/fgeed.2021.663380 (PMC8525372; doi:10.3389/fgeed.2021.663380)
Supplement: Supplementary Table 6 — Primer and probe sequences used in the study and sequences of construct components. [file Table_6.DOCX]

**Supplementary table 6**

| **Copy number determination** | | | |
| --- | --- | --- | --- |
| **Target** | **Forward primer** | **Reverse primer** | **Probe** |
|  |  |  |  |
| mCherry | GAGGCTGAAGCTGAAGGAC | GATGGTGTAGTCCTCGTTGTG | FAM-CCAACTTGATGTTGACGTTGTAGGCG-TAMERA |
| HptII | GGATTTCGGCTCCAACAATG | TATTGGGAATCCCCGAACATC | FAM-CAGCGGTCATTGACTGGAGCGAGG-TAMERA |
| Constans like | TGCTAACCGTGTGGCATCAC | GGTACATAGTGCTGCTGCATCTG | VIC-CATGAGCGTGTGCGTGTCTGCG-TAMERA |

| **PCR primers** | | **Binding site/Purpose** |
| --- | --- | --- |
| F1 | TGCCAAAGTGCTACATCAGC | Flanking left homology arm |
| R1 | AAGCGCATGAACTCCTTGAT | Flanking right homology arm |
| F2 | CACTACGACGCTGAGGTCAA | Internal mCherry |
| R2 | GGCTTGGTGGAGTATGCAGT | Flanking right homology arm |
| R3 | CGTGATGTCATAGCCTGCAT | Flanking right homology arm |
| F4 | CAACGTGGAGACAAGAAGCA | Flanking target site A/indel detection |
| R5 | TCCTTGAGCCGAACCAATATCAC | Flanking target site B/indel detection |

| **Sequencing primers** | | **Purpose** |
| --- | --- | --- |
| Seq1 | TCAGCAGAGGTTAGCCTTTGTAG | Sequencing left junction and full length PCRs |
| Seq2 | CCTGGCGAGTTATTAAGAAAACCA | Sequencing left junction and full length PCRs |
| Seq3 | AGCCAGCTCCAGTTTTGTTC | Sequencing left junction and full length PCRs |
| Seq4 | CATCAAGGAGTTCATGCGCTT | Sequencing left junction and full length PCRs |
| Seq5 | GACGGCGAGTTCATCTACAAG | Sequencing full length PCRs |
| Seq6 | CAAGACCACCTACAAGGCCAA | Sequencing right junction and full length PCRs |
| Seq7 | ACCCAGACCCAGCCATGTT | Sequencing right junction and full length PCRs |
| Seq8 | CATCCCACCACCACAGCA | Sequencing right junction and full length PCRs |
| Seq9 | AGCCCTTCTCGTTCGGACATT | Sequencing right junction and full length PCRs |
| Seq10 | TGGTTTTCTTAATAACTCGCCAGG | Sequencing left junction and full length PCRs |
| Seq11 | AACATGGCTGGGTCTGGGT | Sequencing right junction and full length PCRs |

| **Chromosome walking** | | | |
| --- | --- | --- | --- |
| **T-DNA Right border walk** | | **T-DNA Left border walk** | |
| Rb_SP1 | TGAGACCGAGGATGCACATGTG | Lb_SP1 | ATTTCTTGTGTGCAACTCCGGGAA |
| Rb_SP2 | CATGTGACCGAGGGACACGAAGT | Lb_SP2 | GCCGTTTGTTGCCGCCTTTGTACAACCCCAGT |
| Rb_SP3a | AAGTGATCCGTTTAAACTNNNNNNNNNNNNCAGGAT | Lb_SP3a | CCCAGTCATCGTATATACNNNNNNNNNNNNCGTTAT |
| Rb_SP3b | ACGAAGTGATCCGTTTAANNNNNNNNNNNNTGACAG | Lb_SP3b | TATATACCGGCATGTGGANNNNNNNNNNNNACGTAG |

| **Construct assembly** | |
| --- | --- |
| Protospacer A | GTGACCATGGAGGACGTGGT |
| Protospacer B | GACGGCGGCCACGACCTCCA |
| Repair template | gacggcggccacgacctccacggaagcatgtcaaatttgactcaatttattttttgccaagaaattgtcgtgcttgcaaacttaatttgccaccctgacgccaatataaattgccatacaaaatgtttgatttgccatgcctaattttttagcatttttgtttcttttatcattagccattatcttcttctttttaaaatcttatagtatgcaaatctaataagattcctggcgagttattaagaaaaccagattctcattattttttcctttgcaaaaaagagaagattctctctctctcttacaacgattctcattctccggctcaaaaaaaagtttgtttctcattctcttcctgcttaatgcaatcggtattttttttttgaggggaacttaatgcaatcagtagagtgcttgcctcgttgctggaaaaagaattgatgatccatgtgatttaacgagaaaaacaaagtccgcccatggtgcccaatattttaggcccagttgggatggtagaacctgctgctggagccagctccagttttgttcgccgaatgccgagtcccggcgcccagggatggctataaataagcgagctcccgtgtccttgtgtacttgtaaaatctgtgctccctgcccaccgctctcccctcggttcccacgcgccaaaacattccaacgtggagacaagaagcagcatagcgtgacaacgagggagggagccatggacgtgaccatggaggacgtgatggtgagcaagggcgaggaggataacatggccatcatcaaggagttcatgcgcttcaaggtgcacatggagggctccgtgaacggccacgagttcgagatcgagggcgagggcgagggccgcccctacgagggcacccagaccgccaagctgaaggtgaccaagggtggccccctgcccttcgcctgggacatcctgtcccctcagttcatgtacggctccaaggcctacgtgaagcaccccgccgacatccccgactacttgaagctgtccttccccgagggcttcaagtgggagcgcgtgatgaacttcgaggacggcggcgtggtgaccgtgacccaggactcctccctgcaggacggcgagttcatctacaaggtgaagctgcgcggcaccaacttcccctccgacggcccagtaatgcagaagaaaaccatgggctgggaggcctcctccgagcggatgtaccccgaggacggcgccctgaagggcgagatcaagcagaggctgaagctgaaggacggcggccactacgacgctgaggtcaagaccacctacaaggccaagaagcccgtgcagctgcccggcgcctacaacgtcaacatcaagttggacatcacctcccacaacgaggactacaccatcgtggaacagtacgaacgcgccgagggccgccactccaccggcggcatggacgagctgtacaagctccacggcctgatcgagtccatgctctgcgatgacactctcatcggcacgcccgagcccgacgagcacccagacccagccatgttcacggacggcccctgctactccaacggctccgacccgagcagcaccaccacgacgaacccgggcacgcccgtgcagcacgacgacgacctgccgcaggactgcaatcccgagaagggactccggctgcttcacctgctcatggccgccgccgaggcgctctccggcccgcacaagagccgggagctggcacgggtgatattggttcggctcaaggagatggtctccagcaccagcggcaacgctgccgcgtccaacatggagcgcctcgccgcccacttcaccgacgcgctccaggggctcctcgatgggtcccactccgtcgctgggaccagcaggcaggccgcatcccaccaccacagcaccggcgacgtgttgacggcattccagatgctccaggacatgtcgccctacatgaagttcggccacttcaccgcgaaccaggcgatcctggaggcggtggcgggcgaccggcgcgtccacatcccaccacgtcctccatggtcac |
| Extended repair template | ttatataagcgctattgaactttggaacgaacgaaattgctgcacttttcaagtaaacagtttgcggtcattgtggcagctagcgcgcttccgctgccaagatttttcttcttcttgagaacaccttgccaaagtgctacatcagcagaggttagcctttgtagatggtattgggcttatattgggttccatgggcggagtttgtttttcgaacaccagatttataggcatggtaaatcaaatgtttttatggcacatatgtgttcgctgaggatggcaagtttagttaacagagacggcggccacgacctccacggaagcatgtcaaatttgactcaatttattttttgccaagaaattgtcgtgcttgcaaacttaatttgccaccctgacgccaatataaattgccatacaaaatgtttgatttgccatgcctaattttttagcatttttgtttcttttatcattagccattatcttcttctttttaaaatcttatagtatgcaaatctaataagattcctggcgagttattaagaaaaccagattctcattattttttcctttgcaaaaaagagaagattctctctctctcttacaacgattctcattctccggctcaaaaaaaagtttgtttctcattctcttcctgcttaatgcaatcggtattttttttttgaggggaacttaatgcaatcagtagagtgcttgcctcgttgctggaaaaagaattgatgatccatgtgatttaacgagaaaaacaaagtccgcccatggtgcccaatattttaggcccagttgggatggtagaacctgctgctggagccagctccagttttgttcgccgaatgccgagtcccggcgcccagggatggctataaataagcgagctcccgtgtccttgtgtacttgtaaaatctgtgctccctgcccaccgctctcccctcggttcccacgcgccaaaacattccaacgtggagacaagaagcagcatagcgtgacaacgagggagggagccatggacgtgaccatggaggacgtgatggtgagcaagggcgaggaggataacatggccatcatcaaggagttcatgcgcttcaaggtgcacatggagggctccgtgaacggccacgagttcgagatcgagggcgagggcgagggccgcccctacgagggcacccagaccgccaagctgaaggtgaccaagggtggccccctgcccttcgcctgggacatcctgtcccctcagttcatgtacggctccaaggcctacgtgaagcaccccgccgacatccccgactacttgaagctgtccttccccgagggcttcaagtgggagcgcgtgatgaacttcgaggacggcggcgtggtgaccgtgacccaggactcctccctgcaggacggcgagttcatctacaaggtgaagctgcgcggcaccaacttcccctccgacggcccagtaatgcagaagaaaaccatgggctgggaggcctcctccgagcggatgtaccccgaggacggcgccctgaagggcgagatcaagcagaggctgaagctgaaggacggcggccactacgacgctgaggtcaagaccacctacaaggccaagaagcccgtgcagctgcccggcgcctacaacgtcaacatcaagttggacatcacctcccacaacgaggactacaccatcgtggaacagtacgaacgcgccgagggccgccactccaccggcggcatggacgagctgtacaagctccacggcctgatcgagtccatgctctgcgatgacactctcatcggcacgcccgagcccgacgagcacccagacccagccatgttcacggacggcccctgctactccaacggctccgacccgagcagcaccaccacgacgaacccgggcacgcccgtgcagcacgacgacgacctgccgcaggactgcaatcccgagaagggactccggctgcttcacctgctcatggccgccgccgaggcgctctccggcccgcacaagagccgggagctggcacgggtgatattggttcggctcaaggagatggtctccagcaccagcggcaacgctgccgcgtccaacatggagcgcctcgccgcccacttcaccgacgcgctccaggggctcctcgatgggtcccactccgtcgctgggaccagcaggcaggccgcatcccaccaccacagcaccggcgacgtgttgacggcattccagatgctccaggacatgtcgccctacatgaagttcggccacttcaccgcgaaccaggcgatcctggaggcggtggcgggcgaccggcgcgtccacatcccaccacgtcctccatggtcactgtgcgtggactacgacctcgccgagggcatccagtgggcgtccctgatgcaggctatgacatcacgacccgatggcgtgtcgcctccgcacctgcgtatcaccgccatcacgcggagtggcgggggcggcgcgcgggcagtccaggaggccggacggcgcctcgcggccttcgcggggtccatcgggcagcccttctcgttcggacattgccgtctggactcggacgagaggttccggccggcgaccgtcaggatggtcaagggggagacgctcgtggccaactgcatactccaccaagccgcggcgacgaccaccgtcagacggcccaccggctcggtggcgtccttcttgaccggcatggcctctctcggggccaaggtggtgacggtggtggaggaggaa  Guide target sites  Homology arm extensions  Polymorphisms not expected in GT events  Left homology arm  mCherry  Right homology arm |
| LIR | agtagcaagcagaagcccggcaggtccttagcgaaaaaacggggtgtgctcgcgaactctactctctaccctgcgtgggagtgtgcagaattcacaccgatgggctcggtgtccacggtttaaatattgcaggtttaggtgggaacgcgggaccctgtcttttcggcgcgaaagcgacgggtggtcccgcgtgtggtttgtggctcggggacccgccacgcaggaatctaatattaccctgcgtggcgggtcccgaggcgcactcggcttttcgtgagtgcgccgaggcttttggaccacgtctttatgtcatcacatcaattattggtggtgagtcatcacatattccacctgcaattatgtgccatcgcttagcttataaggaagtgtcggggaaggtatctcg |
| SIR-REP-LIR | acggagtggatgaacacgggtgacggcaagataggcgatattaagaagggtgccctgtatctagtaacctgtactcgtggaggtatcactggagacagtgcctccatttcattcgaagttgtatgtgcctatacgcacgcgtgttacttcaaatccattgggattcaataaataaaatagtattttattcatctcatgtcattcgattacagatgctcggctacgagcaaagataaaccaaactatgacatacaacacactcataaccaaaacatcgaaaaaagaaatacaaggggcgagatcacacaattttagaaaccgtagccgtccgcgctaggacagtcactgcgaagcagtgacattttcgccgaaggcgaagaatgattcaccctcatacatataatgtatcacagcgttagagtacatgtaatccgactgttcaggagtcatatccttgagccaatcttcgtctgggttaactaaaatgatgcaaggtataccaccccgtatcatttttcgcttcccgtacttaggattgacggtgaagtcacgctgagccccgacgaagcacttccagtttggcgtgaacttgaatggaatgtcgtcaattatgttgtacttggcgttgacgtcataggtcgtgaaatcaactaggctgttgtagtagttgtggatccctagagatcttgcccaggaagtctttcctgttcttgtcggaccgcagatgtagatggacttatgccgtcccggtgactcctggaataatcgtccatccactctaaatcagttacggccttatccgcaggagttgaagtacaaaggatatatgattcgaggcttacggagtagagatgttcatttttccagctttcaatggtctcatgacacatgagggactcgatgggaaactcaggtgtgtaagtgctaactgggtctgggaataggtggcgtgcagtgtattcgaagtctttcagacggatagaccattcaaacggaaaacgatggcagaccatgctgagaaattcctctctcgaggtactcgactcaatgatctgtttcatatccgcgtctcggtctttacgacccggagtggtaactgctacgaatgttccccactcagccgtgttgacatcggagtcaacctccttcatgatgtaatcacgaacttggttgcagtctttggcagcttgaatgttaggatgaaaaaatgaaaatggtgatgtgttcataccaatgttgagagcattgggattggtgatggaagcacgaagcttgttttgcacgagtacgtgcagatgtggtgatccatcttcgtggagttccctaactgcagctatgtacagaggttcatatttggccaagagagtgcgaagagagtccaaggcgtactgtggctctaggatgcattgaggatatgttaggaagaggtatttggaatagacacggaacctgggtgcagatgaagaggccatagtagcaagcagaagcccggcaggtccttagcgaaaaaacggggtgtgctcgcgaactctactctctaccctgcgtgggagtgtgcagaattcacaccgatgggctcggtgtccacggtttaaatattgcaggtttaggtgggaacgcgggaccctgtcttttcggcgcgaaagcgacgggtggtcccgcgtgtggtttgtggctcggggacccgccacgcaggaatctaatattaccctgcgtggcgggtcccgaggcgcactcggcttttcgtgagtgcgccgaggcttttggaccacgtctttatgtcatcacatcaattattggtggtgagtcatcacatattccacctgcaattatgtgccatcgcttagcttataaggaagtgtcggggaaggtatctcg |
